# Observations from Mortality Trends at The Children’s Hospital, Accra, 2003-2013

**DOI:** 10.1371/journal.pone.0167947

**Published:** 2016-12-15

**Authors:** Edem M. A. Tette, Margaret L. Neizer, Mame Yaa Nyarko, Eric K. Sifah, Isabella A. Sagoe-Moses, Edmund T. Nartey

**Affiliations:** 1 Princess Marie Louis Children’s Hospital (PML), Accra, Ghana; 2 Department of Community Health, School of Public Health, University of Ghana, Legon, Ghana; 3 Ghana Health Service, Family Health Division Private Mail bag, Ministries, Accra, Ghana; 4 Centre for Tropical Clinical Pharmacology & Therapeutics, School of Medicine and Dentistry, University of Ghana, Accra, Ghana; University of British Columbia, CANADA

## Abstract

**Objective:**

Facility-based studies provide an unparalleled opportunity to assess interventions deployed in hospitals to reduce child mortality which is not easily captured in the national data. We examined mortality trends at the Princess Marie Louise Children’s Hospital (PML) and related it to interventions deployed in the hospital and community to reduce child mortality and achieve the Millennium Development Goal 4 (MDG 4).

**Methods:**

The study was a cross-sectional review of data on consecutive patients who died at the hospital over a period of 11 years, between 2003 and 2013. The total admissions for each year, the major hospital-based and population-based interventions, which took place within the period, were also obtained.

**Results:**

Out of a total of 37,012 admissions, 1,314 (3.6%) deaths occurred and admissions tripled during the period. The average annual change in mortality was -7.12% overall, -7.38% in under-fives, and -1.47% in children ≥5 years. The majority of the deaths, 1,187 (90.3%), occurred in under-fives. The observed decrease in under-five (and overall) mortality rate occurred in a specific and peculiar pattern. Most of the decrease occurred during the period between 2003 and 2006. After that there was a noticeable increase from 2006 to 2008. Then, the rate slowly decreased until the end of the study period in 2013. There was a concomitant decline in malaria mortality following a pattern similar to the decline observed in other parts of the continent during this period. Several interventions might have contributed to the reduction in mortality including the change in malaria treatment policy, improved treatment of malnutrition and increasing paediatric input.

**Conclusion:**

Under-fives mortality at PML has declined considerably; however, the reduction in mortality in older children has been minimal and thus requires special attention. Data collection for mortality reviews should be planned and commissioned regularly in hospitals to assess the effects of interventions and understand the context in which they occur. This will provide benchmarks and an impetus for improving care, identify shortfalls and ensure that the gains in child survival are maintained.

## Introduction

Under-five mortality has received immense public health attention in recent years because many of the causes of these deaths are easily preventable or treatable [1,2]. The fourth Millennium Development Goal (MDG 4) aimed at reducing mortality in children under five years by two thirds between 1990 and 2015 [3]. Over the years, under-five mortality reduced from 12 million in 1990 to approximately 6.9 million in 2011 worldwide and is now mostly concentrated in sub-Saharan Africa [3,4].

Interventions ranging from immunisation programmes, to Integrated Management of Neonatal and Childhood Illnesses (IMNCI) are reported to have contributed to child survival world-wide [4–6]. Others include hospital-based interventions such as training in the management of acute malnutrition, development of case management protocols, introduction of the WHO pocket book for hospital care of sick children, Emergency Triage Assessment and Treatment (ETAT) and Quality Assurance programmes [7–10]. Information on the effects of these interventions on mortality in hospitals is limited.

We determined mortality trends at Princess Marie Louise Children’s Hospital (PML) against a background of interventions to reduce child mortality in order to examine the changes in mortality in under-fives and older children over an 11 year period from 2003–2013. We also examined mortality from malaria during this period.

## Methods

### Study area and setting

The study was carried out at the Princess Marie Louise Children’s Hospital (PML) also known as “The Children’s Hospital” in Accra, Ghana. The hospital, a 74 bed facility with approximately 70,000 out-patient department visits annually, has the largest nutritional rehabilitation centre in the country. Admissions for malnutrition ranged from 2.4% to 7.3% of the total annual admissions from 2005 to 2013. It is the second largest Paediatric facility in the capital. PML serves as a primary and secondary care facility. Parents can self-refer their children to the hospital and referrals are received from other health facilities. Children between the ages of 0 and 17 years are seen there in consonance with the definition of a child in Ghana (Children’s Act 560, 1998, Ghana) [11]. Though neonatal referrals are received from nearby maternity homes, most of these facilities preferred to refer their patients to Korle Bu Teaching Hospital which has a large Neonatal Intensive Care Unit as well as a Special Care Babies Unit and is only 15–30 minutes drive from PML.

Prior to 2005, ill children needing observation at PML were detained in a recovery ward during the day and referred to other facilities if they were too ill to be kept overnight. However, from 2005, an emergency unit was created and ill patients were treated there overnight. This policy was strengthened by recruitment of two diploma holders in Paediatrics in 2006. It is believed that keeping ill children in the hospital increased awareness and use of the hospital which affected both admissions and mortality. Although notable physicians like Dr Cecily Williams, who described Kwashiorkor in detail in 1933, have worked at PML, there was no paediatric specialist working at the hospital just before 2003.

Between 2003 and 2006, admission to the hospital was free for malnourished children. They received a supply of food from the Catholic Relief Services and monetary support from a catholic priest, Father Andrew Campbell and from the income generating activities of the hospital. An NGO also provided training in income generating activities for their mothers but most of this support has waned. Currently, the hospital operates a Needy Children’s Fund and the National Health Insurance Scheme (NHIS). The fund is made up of contributions from private donors and available to children on admission in need of funds to pay for investigations, drugs and emergency care for the first 48 hours. It started in August 2010 and from 2011 to 2013, 365 children had benefitted from the fund. The NHIS was established in 2003 to make health care more accessible to all, and free in some respects [12]. It was part of social protection measures to reduce the effects of poverty (MDG 1) [13,14]. Fees are fully or partially waived for children registered under the scheme. However, parents are required to pay a premium and register themselves before a child can benefit.

### Study design

The study was a descriptive cross-sectional study involving a retrospective review of data on consecutive children who died while on admission at PML from 1^st^ January, 2003 to 31^st^ December, 2013. A list of population-based and hospital-based interventions occurring at the time was compiled, and a literature review was undertaken to identify the expected effects of these interventions. The study was part of a bigger child mortality investigation at the hospital which also looked at the causes of death, the main diagnostic categories and the place of residence and mortality [15,16]. We examined clinical characteristics in more detail and risk factors for mortality in a case-control study which compared children who died in 2011 with children who survived in the same year [17].

### Study population

The study population was made up of children aged 0–17 years who were admitted to the hospital from 1^st^ January, 2003 to 31^st^ December, 2013. Children who died at the hospital during this period were included in the study. The mortality events in this study include all patients who died in the emergency room and wards within the hospital. Patients who were brought-in-dead (BID) or dead on arrival (DOA) were excluded as they were not normally included in the hospital’s mortality data. However, occasionally, one or two may be inadvertently included. PML did not routinely collect data on post-discharge mortality so a record of this data was also not included.

### Data collection methods and instruments

Printouts from computerised and hand written records on all deaths occurring from 2003 to 2013 were obtained from the records department. Data on the age, sex, cause of death, date and time of admission and death and place of residence of patients were entered into a computerised record form by trained data management personnel. The total number of admissions at the hospital for each year was also obtained to enable the deaths to be expressed as a proportion of the admissions. Data on child deaths occurring in June 2003, February 2007 and March 2007 could not be located so they were not included in the analysis. Information on interventions to reduce child mortality nationally and locally was obtained from the Reproductive and Family Health Division, Ghana Health Service, some national programmes and the hospital administration. More details on the data collection process has been extensively described elsewhere [16].

### Data analysis

The data was entered, cleaned and managed using Microsoft Access (Microsoft Corporation, Edmond, Washington) and analysed using Stata SE 11.0 (Stata Corporation, College Station, Texas). Frequencies and proportions were used to describe data variables and they were reported in tabular and graphical forms. Mortality ratios were calculated using data on the number of deaths per 1000 age-specific admissions. Average annual percentage change in mortality ratio was calculated using a 2-year moving average. Each of the age-specific mortality ratios over the 11-year period was reported as an average annual percentage change.

### Ethics

Ethical Clearance was obtained from the Ghana Health Service Ethical Review Committee [ID No.: GHS-ERC: 05/07/12]. We could not obtain consent from the patient’s caregivers. However, patient information was anonymized and de-identified prior to the analysis.

## Results

During the period of the study, out of 37,012 total admissions, 1,314 deaths occurred giving a death rate of 3.6% (range 2.6% to 6.3%). During this period, the admissions tripled from 1,584 in 2003 to 4,727 in 2013 (3 fold increase). Data on child deaths occurring in June 2003, February 2007 and March 2007 could not be located so they have not been included in the analysis. Over half of the deaths occurred in male patients, 52.2% (686). The age range was 1 day to 15 years. The majority of the deaths, 1,187 deaths (90.3%), occurred in under-fives while 127 deaths (9.7%) occurred in older children. Table 1, contains information on patient attendance at PML and socio-economic events during the study period. Table 1, also shows the number of admissions for malaria in relation to all other admissions during the study period. Admissions for malaria and other conditions rose gradually until 2008 when the rise became even steeper. This was followed by a decline in 2012 which was steeper for malaria admissions. Table 1 also shows that the proportion of admissions to outpatients was 2.3% in 2003, it increased to 2.6 in 2005 and was 6.8% in 2013. Table 2 shows the yearly proportionate deaths per 1000 admissions.

**Table 1:** Patient attendance at PML & socio-economic events

Indicators	2003	2004	2005	2006	2007	2008	2009	2010	2011	2012	2013
Total admissions at PML	1584	1970	1940	2157	2589	3237	3805	4519	5283	5201	4727
Malaria admissions at PML	574	555	677	695	1050	1130	1605	1805	1813	1358	1181
Proportion of malaria admissions per total admissions at PML (%)	36.2	28.2	34.9	32.2	40.6	34.9	42.2	39.9	34.3	26.1	25.0
Non-malaria admissions at PML	1010	1415	1263	1462	1539	2107	2200	2714	3470	3843	3546
Discharges at PML	1485	1851	1863	2095	2486	3095	3667	4365	5144	5043	4604
Deaths at PML	99	119	77	62	103	142	138	154	139	158	123
Total OPD Attendance at PML	69399	67757	73955	66975	65766	70835	68983	70911	75288	75900	69482
NHIS OPD %	-	-	-	5	18	33	23	31	42.3	48.1	53.1
NHIS Admissions%	-	-	-	2	8.7	16.9	13.2	15.0	22.5	33.9	40.3
GDP Growth Rate^1^	5.6	5.9	6.2	4.3	9.1	4.8	7.9	14.0	9.3	7.3	4.2
Inflation^2^	29.8	18.2	15.5	11.7	10.7	16.5	19.3	10.8	8.7	9.2	10.2

^1^GDP (Gross Domestic Product) growth rate at constant 2006 prices

^2^Inflation—Percentage Change over 12 months ("Year-on-Year Inflation")

NHIS = National Health Insurance Scheme; OPD = Out-Patients Department; Source of GDP and inflation figures: Ghana Statistical Service

**Table 2:** Yearly proportionate death per 1000 age-specific admissions in PML Children's Hospital in Accra, Ghana, 2003–2013.

	< 5 years		5 years and above		All ages	
Year	Number of patients admitted	Number of deaths, proportion per 1000 age-specific admissions	Number of patients admitted	Number of deaths, proportion per 1000 age-specific admissions	Number of patients admitted	Number of deaths, proportion per 1000 admissions
2003^1^	1450	95 (65.5)	134	4 (29.9)	1584	99 (62.5)
2004	1768	112 (63.3)	202	7 (34.7)	1970	119 (60.4)
2005	1749	72 (41.2)	191	5 (26.2)	1940	77 (39.7)
2006	1951	55 (28.2)	206	7 (34.0)	2157	62 (28.7)
2007^2^	2272	94 (41.4)	317	9 (28.4)	2589	103 (39.8)
2008	2796	119 (42.6)	441	23 (52.2)	3237	142 (43.9)
2009	3386	119 (35.1)	419	19 (45.3)	3805	138 (36.3)
2010	3936	137 (34.8)	583	17 (29.2)	4519	154 (34.1)
2011	4677	130 (27.8)	606	9 (14.9)	5283	139 (26.3)
2012	4621	141 (30.5)	580	17 (29.3)	5201	158 (30.4)
2013	4178	113 (27.0)	549	10 (18.2)	4727	123 (26.0)
**Total**	**32784**	**1187 (36.2)**	**4228**	**127 (30.0)**	**37012**	**1314 (35.5)**

^1^Mortality figures for June 2003 were missing

^2^Mortality figures for February and March 2007 were missing

Missing mortality figures and their corresponding admission figures were not used in calculating proportional deaths per admissions.

Prior to 2005, routine information on neonatal mortality was not collected separately. When collection started in 2005, the information was collected as mortality in children dying in less than a full calendar month, that is, 28 to 31 days. Using this information, there were 98 deaths (8.9% of total deaths) in children less than one month olds giving a death rate of 0.29% of total admissions.

**Fig 1:**
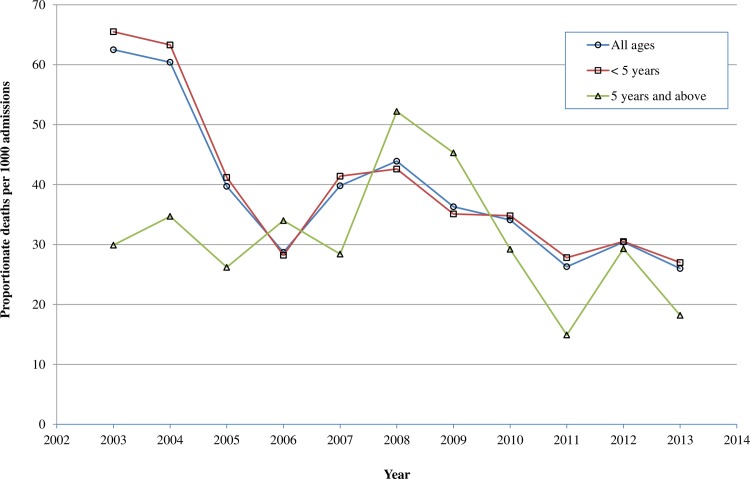
Yearly proportionate death per 1000 admissions across different age categories at PML Children's Hospital in Accra, Ghana, 2003–2013.

Fig 1 shows the trend for yearly proportionate deaths per 1000 age-specific admissions. The mortality trend in both under-fives and older children fluctuated but they generally followed a downward trend from 2003 to 2013 with the under-fives making more progress over the period of 11 years (Fig 1). There was an increase in mortality in both groups from 2006 peaking in 2008 before decreasing with a steeper fall in older children by 2011. A rise in mortality in both groups also occurred from 2011–2012 followed by a fall in 2013. The Average Annual Percentage Change in Mortality was -7.12% for all deaths indicating an average annual decrease. It was -7.38% in under-fives and -1.47% in children aged five years and above.

**Fig 2:**
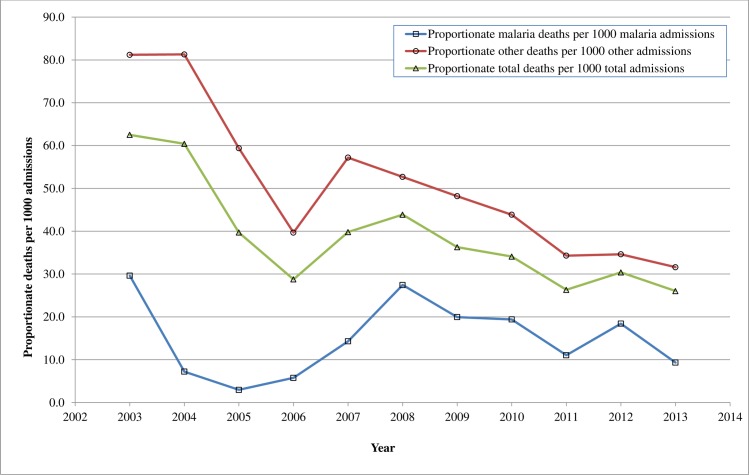
Proportionate malaria deaths per 1000 malaria admissions in comparison with other deaths in PML Children's Hospital in Accra, Ghana, 2003–2013.

**Fig 3:**
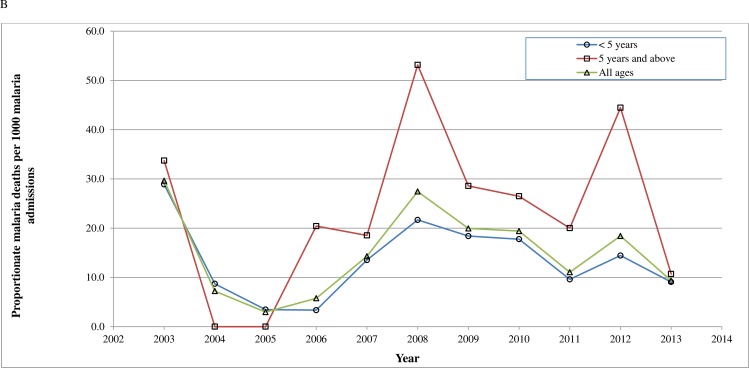
Proportionate malaria deaths per 1000 malaria admissions across different age categories in PML Children's Hospital in Accra, Ghana, 2003–2013.

Figs 2 and 3, show a comparison of the proportion of malaria deaths with other deaths (Fig 2) and malaria deaths per 1000 malaria admissions by age groups (Fig 3) during the study period. Fig 2 shows that there was a sharp decline in both malaria and other deaths between 2003 and 2006 followed by a gradual increase in mortality up to 2007 for malaria and 2008 for the other diseases and then a fall. Fig 3 shows that the decline in malaria from 2003 to 2006 occurred in both under-fives and children aged five years and above. This was followed by a gradual rise with two peaks in 2008 and 2012 evident in both age groups. Diagnosis of malaria was done both clinically and with laboratory tests. Due to a change in the reporting requirement for malaria deaths, only laboratory confirmed malaria cases who died were reported in 2013 whereas both clinically diagnosed and laboratory confirmed cases were reported in the previous years.

Table 3 provides a summary of the various interventions, their duration and their reported effects at both the hospital level (Hospital-based interventions) and national level (Nationwide interventions) and their expected effects. Among the interventions to reduce child mortality provided at the national level was the introduction of the pentavalent vaccine in 2002, consisting of two new antigens, Hemophilus influenza b (Hib) vaccine and Hepatitis B vaccine, which were added to the Diphtheria, Pertussis and Tetanus (DPT) vaccine. The National Health Insurance Scheme’s policy was enacted in 2003 and implemented between 2004 and 2005. In addition to these, Integrated Management of Neonatal and Childhood Illnesses (IMNCI) training had been ongoing at the hospital since 2000. At the hospital level, the interventions included the recruitment of four (4) Paediatric Specialists, one in October 2003, two in 2007 and one in October 2008. Two (2) Diploma in Child Health graduates (2006), house officers (2007), medical officers and seven (7) rotating Paediatric nurses (2012) were also recruited (Table 3).

**Table 3:** Interventions at national level & at PML and expected effects

**Hospital based interventions**	**Duration**	**Proportion of deaths prevented (expected)**
1 Doctor with MSc in Paediatrics	March 2001-Oct. 2008	7% reduction in child mortality per unit increase in paediatricians [18] and from 10–18% per week to 6–8% per week [19]; Reduction of total mortality from 80.5 to 70.5 deaths per 1000 admissions [20].
1 Specialist	October 2003 to 2009	
1 Doctor with MSc in Paediatric (Specialist from 2008)	2003 to present	
2 Doctors with Diploma in Child Health	June 2006 for 1–1½ years	
2 Specialists	October 2007 to present	
1 Specialist	October 2008 to present	
Emergency Unit	2005 to present	From 10–18% per week to 6–8% [19]
Housemen Rotations	May 2007 to present	**-**
WHO Pocket Book on Paediatrics	2007 to present	2.4%, using antibiotic guidelines [21]*
HIV Clinic (ARVs)	2007 to present	75% reduction in mortality in < 2 [22]
Medical Officers Rotations	2008 to present	**-**
CMAM Training	2009 to present	55% Case Fatality (SAM) [23]
Special Care Babies Unit	2009 to present	61% survival for VLBW babies [24]
ETAT Training	2010 to present	From 10–18% per week to 6–8% [19]*; From 80.5 to 70.5 deaths per 1000 admissions [20]*
Paediatric Nurses Rotation	2012 to present	**-**
**Interventions at national level**	**Duration**	**Proportion of deaths prevented (expected)**
IMNCI Training	2000; 2004 to Present	33% of all U5M [6], 13% [25]
Penta-valent vaccine (Hib)	2002 to present	4% of U5M [6]
Measles vaccine and measles campaigns	2002, 2006, 2010 and 2013 (yearly)	1% of U5M [6]; 20% reduction in mortality from non-specific effects [26]
Child Health Record Book & Danger signs	2002 to present	3%, 6%,13% of all U5M [6] ^$^
Anaemia Control Strategy	2003 (1 year)	24% reduced risk of death per 1-g/dL increase in Hb [27]
National Health Insurance Scheme	2003 to present	7% decrease in infant mortality, in US [28];46% lower risk of death in Burkina Faso [29]
Antiretroviral drugs for PMTCT	2003 to Present	2% of all U5M [6]
Change in Malaria Treatment protocol	2004 to present	5% [6], & from 29%-16% [30] of U5M
Maternal & Child Health Promotion Week	2004 to present (yearly)	3%, 6%,13% of all U5M [6] ^$^
Vitamin A supplementation for under-fives	2005 to present	2% of U5M [6]
ITN distribution	2005–2007, 2011, 2012	7% of U5M [6]
CMAM (outpatients management)	2008 to present	-
CMAM (Inpatient management)	2009 to present	55% reduction from SAM (CF)[23]
Child Health Policy	2010 to present	-
Rotavirus Vaccine	2012 to present	30–39% reduction in deaths in 1–2 year olds, 29–33% in1-4 year olds [31]
Pneumococcal Vaccine	2012 to present	16% of all-cause mortality [32]
Zinc Supplementation in Diarrhoea	2012 to present	4% of U5M [6]

ACTs = Artemisinin Combination Therapies; ARVs = Anti-retrovirals; CMAM = Community-based Management of Acute Malnutrition in the community; ETAT = Emergency Triage Assessment and Treatment; Hib = Hemophilus influenza b; IMNCI = Integrated Management of Neonatal and Childhood Illnesses; ITN = Insecticide Treated Net; WHO = World Health Organisation; U5M = under-five mortality; SAM = Severe Acute Malnutrition; CF = Case Fatality; VLBW = very low birth weight infant (birth weight less than 1500g (up to and including 1499g)

* as part of multiple interventions

^$^Selected interventions

## Discussion

### Main Findings

Over the 11 year period, there was a decline in the death rate of admissions at PML from 6.3% in 2003 to 2.6% in 2013 with an average death rate of 3.6%, while admissions tripled from 1,584 in 2003 to 4,727 in 2013 (3 fold increase). From 2004 to 2006, the decline was marked, but was followed by an upsurge in mortality from 2006 to 2008 and then a gradual decline. Majority of the deaths (90.3%), occurred in under-fives. Decline in mortality in children aged five years and above was only minimal.

### Strengths and limitations

The study describes mortality trends over an 11-year period using a large total count of the hospital admissions and the number of deaths. It has demonstrated a marked decline in the in-hospital mortality and explores possible explanations for this by reporting the interventions occurring at the time. It has displayed expected effects reported in the literature for comparison. However, the expected impact is sometimes connected with uncertainty and based on projections or limited data from different health settings making interpretation complex [4,6,33]. Additionally, being a descriptive study, limits the ability of such a study to determine causal factors comprehensively. Furthermore, there was insufficient data to calculate the magnitude of the effect of the interventions using time series analysis. Instances of missing data and lack of detail such as the exact date of the commencement of some of the interventions have been highlighted. The severity of illness was also not scored or analysed with the results.

### Consistency with previous findings

The death rate of 3.6% is lower than the death rate of 4.55% at the Korle Bu Teaching Hospital (KBTH) in Accra during the 1980’s [34], 20.5% in Children’s Emergency Room deaths at Ibadan [35], 9.5% in Ibadan in 2005 [36], 5.8% in the Delta State, Nigeria [37], 21% in Mali [38], and 4% in Mozambique [39]. The study at KBTH also showed that 90% of deaths occurred in children aged five years and below [34]. The mortality rate of 3.62% in under-fives was only slightly higher than that of older children which was 3.0% but lower than the under-five mortality rate of 5.7% reported in a study involving four (4) South African hospitals [40]. Mortality in males (52.2%) was higher than in females (47.8%) and similar to other studies in Africa [34,35,39] but it differs from the study in Mali which reported significantly higher case fatality rates among girls [38].

There are several reasons why admissions have tripled (Table 1). They include a change in admission policy, introduction of the NHIS, an increase in the population of the Greater Accra Metropolitan Area, and the growth of an urban slum, Agbobloshie, near the hospital. The creation of an emergency room in 2005 to keep ill patients in the hospital, allowed more patients to be admitted and might have contributed to the fall in mortality by allowing ill-patients to be better treated. This would have prevented patients from being sent home and returning in a worse state with a heightened risk of mortality. The observation is similar to findings from a study in Malawi which also reported a rise in the number of admissions after creating an Emergency Department and a resulting fall in mortality [19]. Nevertheless, it is also possible for the admission of more ill patients to lead to an increase in mortality as occurs in teaching hospitals [34–36]. Studies have shown that introduction of the NHIS led to an increase in patient attendance to health facilities nationwide [13,14]. Similarly, the population of the Greater Accra Region rose by 840,554 from 3,074,291 in 2003 to 3,914, 845 in 2013. Agbobloshie was one of the places of residence which recorded higher mortality among children attending PML [15].

The decline in mortality from 2004 to 2006 could be due to factors including a striking fall in malaria mortality between 2003 and 2005 (Fig 2), improved management of malnutrition and increasing paediatric presence. The cause specific mortalities for malaria and anaemia (a common manifestation of malaria), declined consistently from 2003 to 2006 in under-fives [16]. Malaria reduced from 12.4/1000 to 1.0/1000 admissions while anaemia reduced from 6.9/1000 to 0.5/1000 admission [16]. This period also coincided with a nationwide decline in child mortality from 2004 to 2008 and a policy change in malaria treatment from Chloroquine to Artemisinin Combination Therapies’ (ACT’s) and Quinine in 2004 [12,41]. Officially, the policy was implemented in 2005 though anecdotal evidence suggests that the new drugs were being used earlier. Epidemiologically, the fall in malaria cases is similar in pattern to the fall in malaria morbidity and mortality occurring in sub-Saharan Africa around the same time in response to changes in malaria treatment policy and the use of insecticide treated nets (ITN’s) [30,42–44]. A study in the Gambia highlighted a dramatic drop in malaria deaths by 100% in one hospital and by 90% in another hospital between 2003 and 2007 [42]. Likewise, a study in Kenya reported a reduction in annual malaria mortality rates from 13.2 to 3.7 per 1,000 person-years from 2003 to 2010 in under-fives [30]. Although the use of ITN’s in Ghana might have influenced the general decline in malaria mortality at PML, by 2008, less than 30% of households in Accra owned at least one ITN [12].

Published data from this mortality study showed that mortality from malnutrition declined from 32.4/1000 admissions to 10.3/1000 admissions between 2003 and 2006 [16]. The reduction can be attributed to additional support and the commencement of a locally prepared ready to use therapeutic food (RUTF). This began after a senior doctor from the hospital returned from a Masters course in Tropical Paediatrics in 2004, during which she undertook research on RUTF in Malawi. The contribution of trained paediatricians to promoting child survival is well recognised [8,18,45–47]. The engagement of a paediatrician in October 2003 is also reported to have led to improvements in care since she trained the nurses, setup a blood storage facility for transfusions and an HIV clinic in 2007, similar to interventions such as ETAT, anaemia control and use of antiretroviral drugs (Table 3). The next fall in mortality, occurred between 2008 and 2010 and from 2010 to 2011. The former occurred just after the posting of three specialist paediatricians to the hospital while the latter coincided with the introduction of ETAT training among others. The engagement of the specialists is reported to have led to a better cover during the day, the establishment of a special care babies unit, introduction of the WHO pocket book and ward protocols for managing common diseases, Table 3 [7,47]. Reductions in mortality due to ETAT have also been attributed to involvement of paediatricians (Table 3) [19,20].

Although the NHIS might have contributed to the overall reduction in under-fives mortality, it is unlikely to have had any major effect between 2004 and 2006 because registration of children attending the hospital was low (Table 1) [48]. Similarly, although a Kenyan study found a reduction in the incidence of Hib disease which became apparent 3 years after introduction of the vaccine, we were unable to do the same [49]. Further analysis of our data (unpublished) showed some reduction in cause specific mortality from pneumonia and septicaemia in under 2 year olds during this period but rose thereafter. It could be that the fall was due to other interventions or the non-specific effects of vaccination campaigns (Table 3) and the limitations of this study design did not enable the contribution of Hib vaccine to be adequately determined [26,50]. Between 2012 and 2013, there was a fall in mortality coinciding with introduction of pneumococcal and rotavirus vaccines.

From 2006 to 2008 there was an upsurge in child mortality in both under-fives and older children. While mortality from malnutrition continued to decrease in under-fives, it increased in all other diseases including malaria, anaemia and infections such as HIV, pneumonia, septicaemia and tuberculosis [16]. Mortality from HIV peaked in 2007 at 13.2/1000 admissions coinciding with the establishment of the HIV clinic possibly because the clinic may have attracted very ill patients to the hospital [16]. The proportion of malaria admissions rose slightly during this period. This might have been partly related to a reduction in compliance with the new malaria treatment due to the side-effects observed after its introduction and the difficulties in administering weight-based paediatric doses of the drug to children at home [12]. Concerns about the side-effects led the health ministry to withdraw a particular version of the treatment in December 2005 [51]. Admission of more ill patients, financial accessibility due to the NHIS and economic challenges in 2008, could also have played a role. The rise in mortality from 2011 to 2012 coincides with a rise in malaria mortality and slower rise in malaria admissions while mortality from other diseases remained largely unchanged (Figs 2 and 3). The rise in malaria mortality was more prominent in children aged five years and above and could suggest changing immunity however the trend was not followed in the next year due to the change in reporting of malaria deaths [16,52].

### Implications

This study shows that PML has implemented most of the recommended interventions for improving the quality of paediatric care in hospitals in developing countries and it provides a catalogue of events that can affect outcome [7–9,46,47]. Our observations indicate that the integrated intervetions were useful in reducing child mortality, but we also highlight difficulties in using routine data to assess causality when several interventions are occurring simultaneously without prior design. Nonetheless, it is important to know how other hospitals in Ghana and elsewhere have fared to ascertain the range of achievable outcomes, improve care and for setting bench marks. The findings also suggest that a significant reduction in the mortality of children aged five years and above requires a different set of intervention and underscores the importance of malaria surveillance in hospitals. This will enable drug resistance to be identified early to prevent deaths from suboptimal treatment, particularly in older children [52]. Thus, the emphasis on surveillance in the new WHO guidelines on malaria control is apt [53]. A robust quality assurance system with data collection to adequately assess the effects of interventions is desirable [7,10,47,54,55]. It can be complement by modified forms of Child Death Review Teams [56].

## Conclusions

There has been a significant decline in mortality at PML which was more pronounced in under-fives and admissions have increased. While it appears that better management of malnutrition, changes in the malaria treatment and increasing paediatric specialist presence have played a significant role, there could be others. More efforts are required to reduce mortality in older children and evaluate outcome.

## Abbreviations

ACTs: Artemisinin Combination Therapies
ARVs: Anti-retrovirals
BID: Brought-in-dead
CF: Case Fatality
CMAM: Community-based Management of Acute Malnutrition
DOA: Dead on arrival
DPT: Diptheria, Pertussis and Tetanus
ETAT: Emergency Triage Assessment and Treatment
Hib: Hemophilus influenza b
HIV: Human Immunodeficiency Virus
IMNCI: Integrated Management of Neonatal and Childhood Illnesses
ITN: Insecticide Treated Net
KBTH: Korle Bu Teaching Hospital
MDGs: Millennium Development Goals
NHIS: National Health Insurance Scheme
PML: Princess Marie Louise Children’s Hospital
RUTF: Ready to Use Therapeutic Food
SAM: Severe Acute Malnutrition
U5M: Under-five mortality
VLBW: Very low birth weight infant
WHO: World Health Organisation
